# Retenção das Habilidades de Ressuscitação Cardiopulmonar nos Estudantes de Medicina: O que Fazer para Melhorar?

**DOI:** 10.36660/abc.20210856

**Published:** 2021-11-01

**Affiliations:** 1 Universidade de São Paulo Faculdade de Medicina Hospital das Clínicas São Paulo SP Brasil Instituto do Coração (InCor) do Hospital das Clínicas da Faculdade de Medicina da Universidade de São Paulo, São Paulo, SP – Brasil

**Keywords:** Medicina, Estudantes de Ciências da Saúde, Ocupações em Saúde/educação, Reanimação Cardiopulmonar/métodos, Habilidade, Competência Clínica, Capacitação Profissional

Desde os primórdios grandes pesquisadores trabalhavam para estimar qual seria a melhor técnica para manutenção do fluxo sanguíneo corpóreo de uma vítima em PCR. Foram aplicadas várias técnicas, como por exemplo: Método do trotar e rolamento sobre barril.^1^ A técnica de compressões torácicas externa foi concebida em 1960, a partir da observação feita por Kouwenhoven, Jude e Knickerbocker^2^ de que a compressão sobre o terço inferior do esterno, feita adequadamente, fornecia uma circulação artificial suficiente para manter a vida em animais e seres humanos com parada cardíaca. Desde então, muitos estudos foram realizados a fim de aprimorar qual seria a profundidade e frequência de compressões apropriadas para manter a perfusão coronariana em nível adequado, colaborando para o retorno da circulação espontânea.

De acordo com as publicações das diretrizes mundiais de 2020,^3^ a realização de compressões de alta qualidade refere-se à realização de compressões a uma frequência de 100 -120 por minuto, profundidade de 5-6cm, retornar o tórax a posição normal entre as compressões *minimizar interrupções nas compressões e evitar ventilação excessiva*. Neste sentido, surge uma grande questão: entendemos quais são os parâmetros da realização de boas compressões, que colaboram significativamente no aumento da sobrevivência de vítimas de PCR. Porém, como garantir que profissionais da saúde e público em geral consiga aprender a técnica e reter este aprendizado, a ponto de reproduzi-lo em uma situação real de emergência?

A tecnologia e a simulação para educar a ressuscitação ganharam importância crescente, promovendo mudanças na forma como os treinamentos são realizados, uma vez que o treinamento em simuladores possibilita ao aluno que a mesma técnica seja repetida diversas vezes, desenvolvendo assim a competência necessária.^4,5^

Nesta edição dos Arquivos Brasileiros de Cardiologia, Moretti et al.,^6^ apresentaram um estudo prospectivo caso controle, onde foi avaliado 50 estudantes de medicina em habilidades de suporte básico de vida. Eles foram avaliados no desempenho das habilidades imediatamente após o curso e 06 meses depois. O número de etapas cumpridas de forma correta após seis meses foi significativamente menor que logo após o curso (10,8 vc 12,5 p< 0,001).

A principal questão para reflexão ao ler este artigo é: como manter a retenção do aprendizado das habilidades de RCP? Segundo as diretrizes de educação em ressuscitação da *European Resuscitation Council,*^4^ as habilidades de SBV decaem dentro de 3 a 12 meses após a educação inicial em RCP, mas as competências de ressuscitação são mais bem mantidas se o treinamento e o retreinamento forem distribuídos ao longo do tempo, entre dois e doze meses.

Neste sentido, a tendência atual nos treinamentos em emergências se baseia no novo conceito de “*low-dose and high frequence*” - baixa dosagem e alta frequência, que utiliza uma abordagem de desenvolvimento e promoção da máxima retenção do conhecimento clínico, habilidades e atitudes. O treinamento conta com as atividades de aprendizagem baseadas em simulações curtas e específicas, espaçadas ao longo do tempo e reforçadas com sessões práticas estruturadas e contínuas no local de trabalho.^7^

Outra proposta é a utilização de dispositivos de feedback durante o treinamento de ressuscitação, Estes dispositivos são providos de recursos audiovisuais, que permitem o acompanhamento do desempenho na realização da RCP, em relação a diversos parâmetros, como: frequência e profundidade das compressões, fração de compressão, frequência e volume das ventilações, entre outros.^8^

Uma revisão sistemática publicada em 2021,^9^ sobre a melhoria da qualidade da RCP, utilizando dispositivos de feedback, concluiu que estes dispositivos melhoram a aquisição de habilidades e o desempenho de RCP durante o treinamento de profissionais de saúde.

Assim, a leitura deste estudo nos traz uma reflexão sobre o presente e futuro dos treinamentos em ressuscitação. Mais ainda, como os serviços e universidades de saúde podem implementar melhores práticas educacionais, que levam a melhores resultados para o paciente após a parada cardíaca. Isto é a nossa prioridade máxima: salvar vidas!
