# Supplementary material for: Retenção das Habilidades de Ressuscitação Cardiopulmonar nos Estudantes de Medicina: O que Fazer para Melhorar?
Source: Arq Bras Cardiol. 2021 Nov 1;117(5):1036–7. [Article in Portuguese] doi: 10.36660/abc.20210856 (PMC8682110; doi:10.36660/abc.20210856)
Supplement: Translation

# Translation: Retention of Cardiopulmonary Resuscitation Skills in Medical Students: What Can Be Done to Improve Them?

**Affiliations:** 1 São Paulo SP Brazil Instituto do Coração (InCor) do Hospital das Clínicas da Faculdade de Medicina da Universidade de São Paulo, São Paulo, SP – Brazil

**Keywords:** Medicine, Students, Health Occupational, Health Occupations/education, Cardiopulmonary Resuscitation/methods, Clinical Competence, Professional Training

Since the beginning, great researchers have been working on estimating the best technique for maintaining the body blood flow of a victim undergoing CPA. Several techniques were applied, such as the Trotting method and rolling over a barrel.^1^ The technique of external chest compressions was conceived in 1960, based on the observation made by Kouwenhoven, Jude, and Knickerbocker^2^ that adequate compression on the lower third of the sternum provided sufficient artificial circulation to sustain life in animals and humans with cardiac arrest. Since then, many studies have been carried out to improve the depth and frequency of appropriate compressions to maintain coronary perfusion at an adequate level, contributing to the return of spontaneous circulation.

According to the 2020^3^ worldwide guidelines publications, performing high-quality compressions refers to performing compressions at a frequency of 100 -120 per minute, depth of 5-6cm, returning the chest to the normal position between compression *minimize interruptions in compressions avoiding excessive ventilation*. In this sense, a big question arises: do we understand the parameters for performing good compressions, which significantly increase the survival of CPA victims? However, how to ensure that health professionals and the general public can learn the technique and retain this learning to the point of reproducing it in an actual emergency?

The technology and simulation to educate resuscitation have gained increasing importance, promoting changes in the way training is conducted, as training in simulators enables the student that the same technique be repeated several times, thus developing the necessary competence.^4,5^

In this issue of the Brazilian Archives of Cardiology, Moretti et al.^6^ presented a prospective case-control study, where 50 medical students were evaluated in basic life support skills. They were assessed on skill performance immediately after the course and 06 months later. The number of steps correctly completed after six months was significantly lower than right after the course (10.8 vc 12.5 p<0.001).

When reading this article, the main question for reflection is: how to maintain the retention of learning CPR skills? According to the resuscitation education guidelines of the European Resuscitation Council,^4^ BLS skills decay within 3 to 12 months after initial CPR education. Still, resuscitation skills are better maintained if training and retraining are distributed throughout the period between two and twelve months.

In this sense, the current trend in emergency training is based on the new concept of “low-dose and high frequency” – low dose and high frequency, which uses an approach of developing and promoting maximum retention of clinical knowledge, skills and attitudes. The training relies on learning activities based on short, specific simulations over time and is reinforced with structured and continuous practical sessions in the workplace.^7^

Another proposal is the use of feedback devices during resuscitation training. These devices are provided with audiovisual resources, which allow the monitoring of performance in performing CPR, in relation to several parameters, such as: frequency and depth of compressions, compression fraction, frequency and volume of ventilations, among others.^8^

A systematic review published in 2021,^9^ on improving the quality of CPR using feedback devices concluded that these devices improve CPR skill acquisition and performance during the training of healthcare professionals.

Thus, reading this study brings us a reflection on the present and future of resuscitation training. Furthermore, health services and universities can implement better educational practices that lead to better patient outcomes after cardiac arrest. This is our top priority: saving lives!
